# Item response analysis of the Positive and Negative Syndrome Scale

**DOI:** 10.1186/1471-244X-7-66

**Published:** 2007-11-15

**Authors:** Darcy A Santor, Haya Ascher-Svanum, Jean-Pierre Lindenmayer, Robert L Obenchain

**Affiliations:** 1 School of Psychology, University of Ottawa, Ottawa, Canada, and The Provincial Centre of Excellence for Child and Youth Mental Health, Ottawa, Ontario, Canada; 2 Eli Lilly and Company, Indianapolis, Indiana, USA; 3 New York University School of Medicine, New York, New York, USA

## Abstract

**Background:**

Statistical models based on item response theory were used to examine (a) the performance of individual Positive and Negative Syndrome Scale (PANSS) items and their options, (b) the effectiveness of various subscales to discriminate among individual differences in symptom severity, and (c) the appropriateness of cutoff scores recently recommended by Andreasen and her colleagues (2005) to establish symptom remission.

**Methods:**

Option characteristic curves were estimated using a nonparametric item response model to examine the probability of endorsing each of 7 options within each of 30 PANSS items as a function of standardized, overall symptom severity. Our data were baseline PANSS scores from 9205 patients with schizophrenia or schizoaffective disorder who were enrolled between 1995 and 2003 in either a large, naturalistic, observational study or else in 1 of 12 randomized, double-blind, clinical trials comparing olanzapine to other antipsychotic drugs.

**Results:**

Our analyses show that the majority of items forming the Positive and Negative subscales of the PANSS perform very well. We also identified key areas for improvement or revision in items and options within the General Psychopathology subscale. The Positive and Negative subscale scores are not only more discriminating of individual differences in symptom severity than the General Psychopathology subscale score, but are also more efficient on average than the 30-item total score. Of the 8 items recently recommended to establish symptom remission, 1 performed markedly different from the 7 others and should either be deleted or rescored requiring that patients achieve a lower score of 2 (rather than 3) to signal remission.

**Conclusion:**

This first item response analysis of the PANSS supports its sound psychometric properties; most PANSS items were either very good or good at assessing overall severity of illness. These analyses did identify some items which might be further improved for measuring individual severity differences or for defining remission thresholds. Findings also suggest that the Positive and Negative subscales are more sensitive to change than the PANSS total score and, thus, may constitute a "mini PANSS" that may be more reliable, require shorter administration and training time, and possibly reduce sample sizes needed for future research.

## Background

The Positive and Negative Syndrome Scale (PANSS) is the most widely used measure of symptom severity in schizophrenia [1-3]. This 30-item scale is typically administered by trained clinicians who evaluate patients' current severity level on each symptom (item) by endorsing 1 of 7 options (weights) numbered 1 through 7. The PANSS has demonstrated high internal reliability [4,5], good construct validity [4], and excellent sensitivity to change in both short term [6] and long term trials [7]. However, despite extensive psychometric research, it is unclear how individual PANSS items differ in their usefulness in assessing the severity of schizophrenia.

Indeed, studies examining the psychometric properties of the PANSS have focused, without exception, on estimates of scale reliability, validity, and factor analysis using methods from classical test theory [8] and have typically identified 5 underlying factors [9-14]. Approaches based on classical test theory rely primarily on omnibus statistics that average over levels of individual variation and offer no means to gauge the quality of individual items or options across different levels of symptom severity. In contrast, methods based on item response theory (IRT) [15] provide significant improvements over classical techniques, as they model the relation between item responses and symptom severity directly, quantifying how the performance of individual items and options (severity levels 1 to 7) change as a function of overall, standardized, symptom severity. IRT analyses provide unique and relevant information concerning (a) how well a set of item options assess the entire continuum of symptom severity, (b) whether weights assigned to individual item options are appropriate for measuring a particular trait or symptom, and (c) how well individual items or subscales are connected to the underlying construct and discriminate among individual differences in symptom severity (see the publication by Santor and Ramsay [16] for an overview).

IRT methods are ideal for examining the performance of options within items that are to be used to define remission of psychopathology. Andreasen and her colleagues [17] published guidelines recommending that schizophrenia remission be defined as achieving option scores less than or equal to 3 on each of only 8 PANSS items: Delusions, Unusual Thought Content, Hallucinatory Behavior, Conceptual Disorganization, Mannerisms and Posturing, Blunted Affect, Social Withdrawal, and Lack of Spontaneity. Setting equal remission thresholds (≤3) for all 8 items suggests that the level of symptom severity corresponding to "3 or less" is generally equal for all 8 items. If the region of symptom severity at which Options 1, 2, and 3 are most likely to be endorsed differs across items, then some items are more influential than others in determining whether or not remission has been met. Alternately, if the threshold of "3 or less" corresponds to higher severity for an item (i.e., is more easily achieved as symptom severity improves), then either that item is redundant (since it is more likely to be reached first) or else its threshold should be revised downward. For example, the remission threshold for that item could be set at 2 rather than at 3. IRT analyses examine the manner in which individual item options (and cutoff scores) are related to overall symptom severity which is central to evaluating the appropriateness of proposed cut-point scores (thresholds) determining illness remission.

The 3 primary purposes of our study are (a) to examine and characterize the performance of individual items from the PANSS at both the option (severity) and item (symptom) levels with the goal of identifying areas for improvement, (b) to examine the ability of various subscales to discriminate among individual difference in illness severity, which might then identify a better measure of change, and (c) to evaluate the appropriateness of items and options proposed for determining when symptoms remission has been achieved.

## Methods

### Participants

Data included baseline PANSS item scores from 9205 patients with schizophrenia, schizoaffective, or schizophreniform disorders who were enrolled between 1995 and 2003 in either a large, naturalistic, observational study or else in 1 of 12 randomized, double-blind clinical trials comparing olanzapine to other antipsychotic drugs. Participants' diagnosis was based on DSM-IV criteria per treating physician and patient's medical record. Participants were primarily male (65%), could be either inpatients or outpatients, and had an average age of 39.0 (SD = 11.5). Their initial PANSS total score averaged 81.9 (SD = 22.0) but ranged broadly from 30 to 177.

### Analytic models

IRT consists of a broad class of statistical procedures [18] used to model the association between responses to survey items (in probabilistic terms) and an underlying latent trait, characteristic, or condition, such as overall symptom severity. We employed the same nonparametric kernel-smoothing techniques implemented in the software (TESTGRAF) developed by Ramsay [19,20] to estimate option characteristic curves, which serve as a measure of item and option effectiveness. These techniques have been used previously to examine the psychometric properties of self-report scales and to evaluate item bias [21-23] and a detailed description of the algorithm used to estimate response curves has been published elsewhere [16,19].

**Figure 1:**
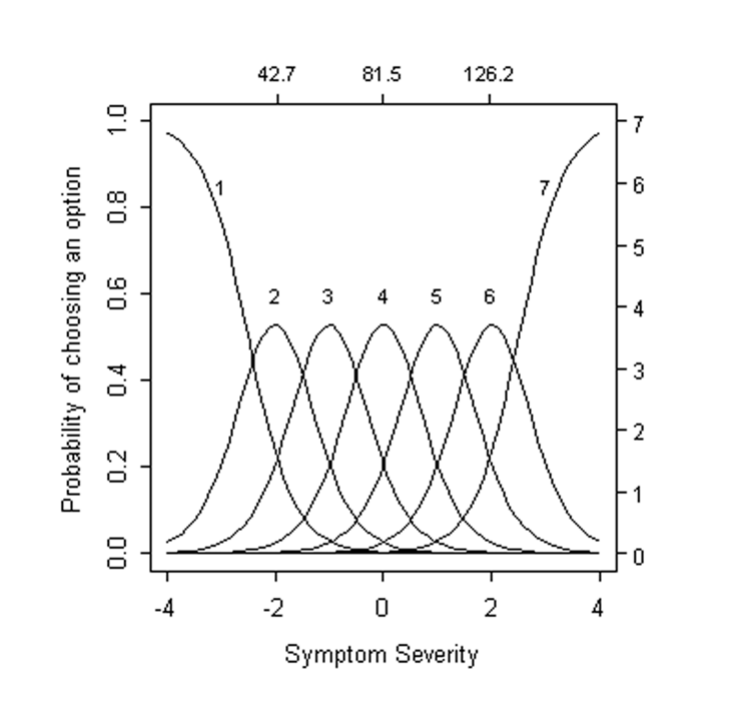
**Option characteristic curves for an ideal item**. The probability of endorsing an individual option (y-axis) from this idealized item is plotted as a function of expected total scores (upper x-axis) and standard normal quantiles (lower x-axis). Expected total scores show the level of symptom severity at which different options are endorsed. Standard normal quantiles, which are analogous to z-scores, show the proportion of the sample at different levels of expected total scores. Key features of this idealized item are described in the main text.

To illustrate how item response models can be used to evaluate item and option performance, we present a set of hypothetical, "ideal" option characteristic curves in Figure 1, where severity has been expressed in both expected total scores (upper x-axis) and standard normal scores (lower x-axis). Expected total scores show the level of symptom severity at which different options are endorsed. Standard normal quantiles, which are analogous to z-scores, show the proportion of the sample at different levels of expected total scores.

Expressing severity this way is useful in that standard normal scores contain rather widely appreciated information about the proportion of a population above or below integer values of -3, -2, -1, 0, +1, +2, and +3. Extreme values on our curves still need to be interpreted with caution, because, in spite of the very large number of PANSS scores evaluated here, sample sizes are small in the tails of the overall severity distribution but still express information about relative levels of symptom severity. We have also superimposed corresponding total symptom scores to facilitate the interpretation of standard normal scores. A standard normal score of -2 corresponds to a total scale score of 37.3 in our sample, and a standard normal score of +2 corresponds to a total scale score of 139.8.

A number of important features of item performance are illustrated in Figure 1. First, the region in which each option tends to be endorsed most frequently is clearly indicated. For example, the curve for Option 2 suggests that the probability of being endorsed is essentially 0 at standardized severity -3, increases to about 0.52 at -1, and then returns to roughly 0 at severity +1. Specifically, Option 2 is more likely to be endorsed than any other option within the overall severity range from -1.5 to -0.5.

Second, each of the option characteristic curves ideally increases rather rapidly with small increases in severity. For example, the probability of Option 2 being endorsed doubles from 0.2 to 0.4 when severity increases by just half of a standard unit from -2.0 to -1.5. Third, the severity regions over which each option is most likely to be endorsed are ordered, left to right, in the same way as the option scores (weights) themselves. That is, the region in which Option 2 is most likely to be endorsed (near -1), falls *between *the regions in which Option 1 (near -2) and Option 3 (near 0) are most likely to be endorsed.

Fourth, together, the options for an item span the full continuum of severity from -3 to +3. Some options are only endorsed at high levels of severity (e.g., Options 5 and 6), whereas others are endorsed at low levels of severity (e.g., Options 0 and 1). If the majority of options on an item are endorsed at low levels of severity, one might characterize that item as too "easy"; a low severity patient might receive a high score on such an item. In contrast, if the majority of options on an item are endorsed only at high levels of severity, that item might be characterized as too "hard"; a high severity patient could receive a low score on such an item. Scales comprised primarily of "hard" items will be largely insensitive to individual differences in the lower or moderate range of symptom severity and produce floor effects. Scales comprised primarily of "easy" items will be largely insensitive to individual differences in the high range of symptom severity and produce ceiling effects.

Once option characteristic curves have been estimated, expected item scores and expected total and subscale scores are easily computed. One simply multiplies the smoothed estimate of probability of endorsing each option by its option weight and then sums these products across all options within each item. Due to variation in endorsement of options within items, these expected scores are arguably better estimates of the true distribution of schizophrenia severity measured by the PANSS than are the corresponding distributions of observed scores and sums. After all, expected item scores can be computed at each point along the severity continuum from -3 to +3.

Using the idealized item portrayed in Figure 1, we judge items as "very good" (a) if there was some range of severity in which the majority of options were more likely to be endorsed than any other, (b) if option characteristic curves increased rapidly with changes in overall severity, (c) if the region in which each option was most likely to be endorsed were ordered, left to right, in accordance with their option scores (weights), and (d) if options for an item spanned the full continuum of severity from -3 to +3. Items were judged as "good" if they had many, but not all, of these features and "poor" if they showed few, if any, of these criteria. We recognize that these ratings are global assessments of 4 very different criteria. Global ratings of "very good", "good", and "poor" along with ratings for each of these 4 criteria for each of the 30 PANSS items are recorded in Table 1. We provided the criteria to 2 blinded independent raters who were able to reproduce a similar pattern of results.

**Table 1:** Discrimination Effectiveness for the PANSS Items

Item	Content	Summary Evaluation	3 Factor Subscales	Criterion A	Criterion B	Criterion C	Criterion D
1.	Delusions	Very good	Positive	6	Yes	Yes	Yes
2.	Conceptual Disorganization	Very good	Positive	6	Yes	Yes	Yes
3.	Hallucinatory Behavior	Very good	Positive	4	Some	Yes	Yes
4.	Excitement	Good	Positive	6	Yes	Yes	No
5.	Grandiosity	Good	Positive	4	Some	Yes	Yes
6.	Suspiciousness/Persecution	Very good	Positive	6	Yes	Yes	Yes
7.	Hostility	Poor	Positive	6	Yes	Yes	No
8.	Blunted Affect	Very good	Negative	6	Yes	Yes	Yes
9.	Emotional Withdrawal	Very good	Negative	7	Yes	Yes	Yes
10.	Poor Rapport	Good	Negative	6	Yes	Yes	Yes
11.	Passive Apathetic Social Withdrawal	Very good	Negative	7	Yes	Yes	Yes
12.	Difficulty in Abstract Thinking	Poor	Negative	6	Some	Some	Yes
13.	Lack of Spontaneity Conversation	Very good	Negative	6	Yes	Yes	Yes
14.	Stereotyped Thinking	Poor	Negative	6	No	Some	Yes
15.	Somatic Concern	Poor	General	4	No	Some	No
16.	Anxiety	Good	General	5	Yes	Yes	No
17.	Guilt Feelings	Good	General	5	Some	Yes	No
18.	Tension	Good	General	5	No	Yes	Yes
19.	Mannerisms and Posturing	Poor	General	6	Some	Yes	Yes
20.	Depression	Poor	General	5	Yes	Yes	No
21.	Motor Retardation	Good	General	5	Yes	Yes	No
22.	Uncooperative	Good	General	6	Yes	Yes	No
23.	Unusual Thought Content	Poor	General	6	Yes	Yes	No
24.	Disorientation	Poor	General	4	No	No	No
25.	Poor Attention	Poor	General	5	Yes	Yes	Yes
26.	Lack of Judgment and Insight	Poor	General	5	Some	Some	Yes
27.	Disturbance of Volition	Poor	General	6	Yes	Yes	Yes
28.	Poor Impulse Control	Good	General	6	Yes	Yes	No
29.	Preoccupation	Poor	General	5	Some	No	Yes
30.	Active Social Avoidance	Very good	General	6	Yes	Yes	Yes

Criterion A: Number of options for which there was some range of symptom severity in which the question was more likely to be endorsed than all other options.

Criterion B: Extent to which option characteristic curves increased rapidly with changes in overall severity.

Criterion C: Does the severity region in which each option was most likely to be endorsed correspond to option weights?

Criterion D: Do options for an item span the full continuum of severity from -3 to +3?

### Defining a continuum of symptom severity

Given that option characteristic curves depend on how symptom severity is defined, we confirmed the appropriateness of modeling of items via their subscale scores by conducting principal components analyses on our sample of 9205 responses on all 30 PANSS items using varimax rotation and Kaiser Normalization prior to modeling. Although the PANSS was originally designed with 3 subscales (Positive, Negative, and General Psychopathology), recent studies examining the internal structure of the scale [9-14] have typically identified 5 underlying factors: (a) positive symptoms, (b) negative symptoms, (c) hostile symptoms, (d) disorganized symptoms, and (e) symptoms of anxiety and depression. Our results suggest a 6-factor solution, with the first 5 factors corresponding to the solution identified by Davis and Chen [9]. Using both the "proportion equal to 1" and the "eigenvalue greater than or equal to 1" criteria, 2 of the 10 items assessing disorganized symptoms, namely Items 12 (Difficulty in Abstract Thinking) and 24 (Disorientation), loaded greater than 0.30 on a sixth factor. Eigenvalues for the 6 factors were 4.56 (Negative Symptoms), 3.41 (Disorganized Symptoms), 3.16 (Positive Symptoms), 2.83 (Hostility Symptoms), 2.48 (Anxiety and Depression Symptoms), and 1.65 (Items 12 and 24). Given that the internal consistency coefficient for the 10 disorganized symptoms (including Items 12 and 24) was still good (Cronbach α = 0.83), we elected to retain a 5-factor solution and to include Items 12 and 24 as part of the Disorganized Symptoms subscale, which was originally identified by Davis and Chen [9] and independently validated by others [11,13,14,24].

Cronbach α's for the 5 subscales were (a) positive factor, 0.85, (b) negative factor, 0.86, (c) hostile factor, 0.78, (d) disorganized factor, 0.83, and (e) anxiety and depression factor, 0.70. However, we also examined the performance of items that comprise the original General Psychopathology subscale when comparing the ability of subscales to discriminate among individual differences in illness severity. Internal consistency for the original General Psychopathology subscale was also adequate, 0.88, suggesting that any of the original or newly recommended subscales would be meaningful indicators of underlying symptom severity against which individual items and options could be modeled.

## Results

Our results are grouped in 3 categories: (a) item response modeling of individual items and options from the PANSS as a function of symptom severity, (b) item response modeling of various subscales, and (c) item response modeling of cutoff scores used to establish symptom remission.

### Examining individual items and options from the PANSS

See Additional file 1 which presents option characteristic curves for all items. A select number of option characteristic curves for items from the various subscales are presented here and discussed in detail, including overall performance as well as proposed remission thresholds.

#### Delusions

**Figure 2:**
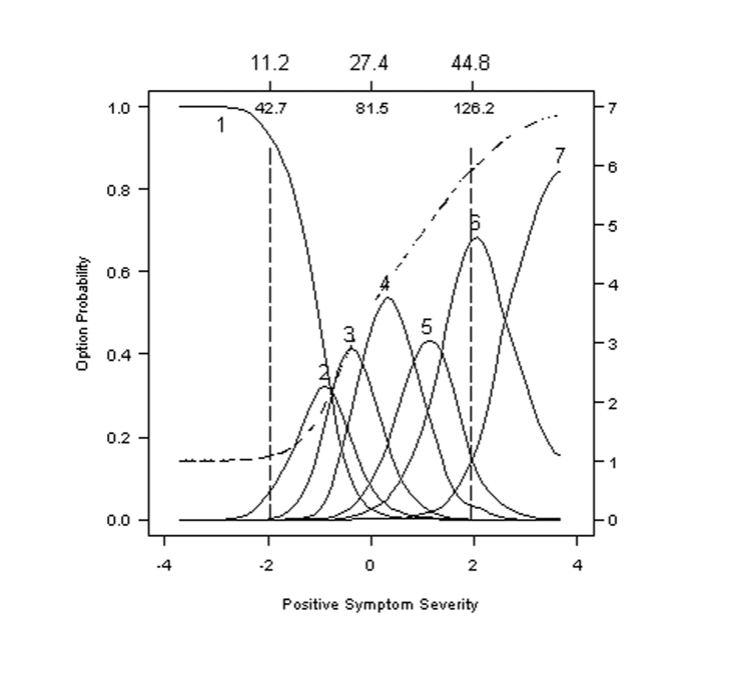
**Option characteristic curves and expected item total score for Item 1: Delusions**. Option characteristic curves (solid lines) and expected item total score (dashed line) are plotted as a function of scores on the Positive Symptomatology Subscale form the PANNS, expressed as standard normal scores (lower x-axis) and expected total scores (upper x-axis). The probability of endorsing an option characteristic curve (solid lines) is scaled on the left y-axis and the expected item score (dashed line) is scaled on the right y-axis. Many features of an ideal item are evident in this plot.

Item 1, from the PANSS Positive Factor subscale, assesses suspiciousness, defined as "Beliefs which are unfounded, unrealistic, and idiosyncratic." Option characteristic curves for this item are presented in the second panel of Figure 2 and show that the probability of rating Option 1, assessing an "absence of symptoms," decreases rapidly as the severity of positive psychotic symptoms begins to increase. Meanwhile, the probability of endorsing the more severe levels of Delusions begins to increase rapidly with increases in the severity of positive symptoms.

Option 2 assessing a "minimal level of suspiciousness" is most frequently endorsed when severity reaches a standard normal score of -1, which corresponds to an expected total score of about 19.0 on the Positive Factor subscale and a PANSS total of 60 and then quickly decreases with further increases in severity. Other option characteristic curves on this item perform equally well. Note that 6 out of the 7 response options (all but 2) have a region where it is more likely to be endorsed than any other option, and all 7 regions occur in the same order as their weights. For example, the region in which Option 3 of mild symptomatology (i.e., "Presents a guarded or even openly distrustful attitude, but thoughts, interactions, and behavior are minimally affected.") is most likely to be endorsed lies between the region in which Option 2, "Questionable pathology", and Option 4, "Moderate" levels of psychopathology, are most likely to be endorsed.

Examining the shape of the curves also provides useful information regarding the precision with which individuals can be designated as showing mild or moderate psychopathology. Results show that, despite well-differentiated peaks for Options 3 (<0) and 4 (>0) on Item 1 (Delusions), the response characteristic curves for these options overlap considerably suggesting that designations made solely on the basis of this question could be imprecise and unreliable.

#### Hallucinatory behavior

**Figure 3:**
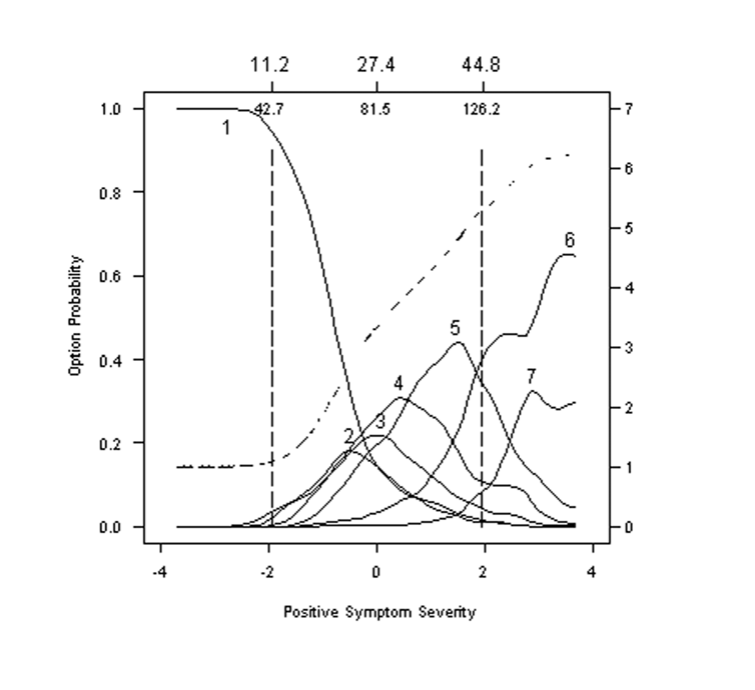
**Option characteristic curves and expected item total score for Item 3: Hallucinatory behavior**. Option characteristic curves (solid lines) and expected item total score (dashed line) are plotted as a function of scores on the Positive Symptomatology Subscale form the PANNS, expressed as standard normal scores (lower x-axis) and expected total scores (upper x-axis). Option characteristic curves (solid lines) indicate that a number of opportunities for improvement. Options 2, 3 and 4 overlap substantially as do Options 6 and 7 suggesting rating these options is inherently difficult for raters.

It is instructive to compare the option characteristic curves for Item 1, Delusions, in Figure 2 with those for Item 3, Hallucinatory Behavior, defined as "Verbal report [s] or behavior indicating perceptions which are not generated by external stimuli" in Figure 3. Options characteristic curves for Item 3 are far less differentiated than those for Item 1. Note that Option 4 of Item 3 entirely overlaps Options 2 and 3. Indeed, at all levels of severity, the probability of endorsing Option 4 is equal to or greater than the probability of endorsing Options 2 or 3.

This level of analysis allows scale developers to identify items and options that might be improved either through training of raters or revision of options/items.

#### Grandiosity

**Figure 4:**
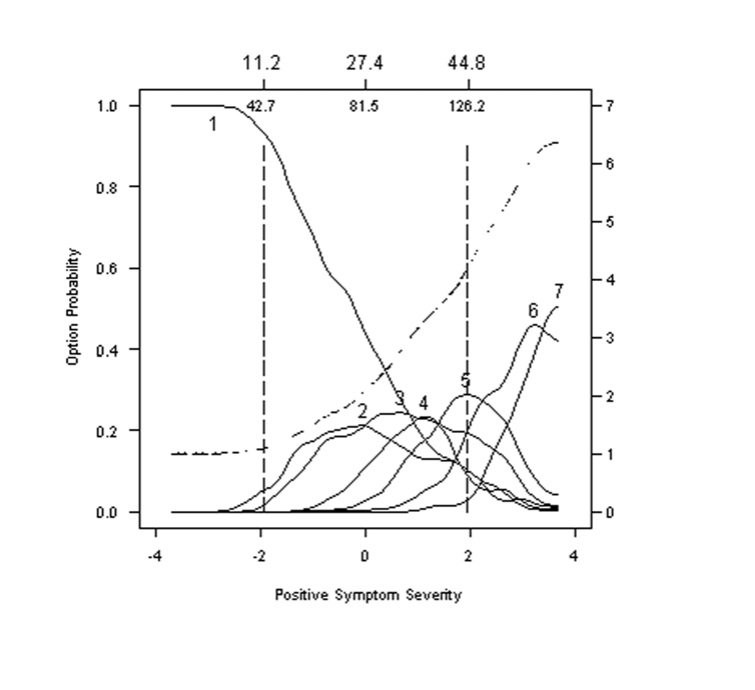
**Option characteristic curves and expected item total score for Item 5: Grandiosity**. Option characteristic curves (solid lines) and expected item total score (dashed line) are plotted as a function of scores on the Positive Symptomatology Subscale form the PANNS, expressed as standard normal scores (lower x-axis) and expected total scores (upper x-axis). Option characteristic curves (solid lines) indicate that a number of opportunities for improvement. Options 2, 3 and 4 overlap substantially as do Options 6 and 7 suggesting rating these options is inherently difficult for raters. Option 1 in this item was most frequently endorsed by over half the sample, indicating that these options are only endorsed as a higher level of symptoms severity than in other options. The item characteristic curve (broken line) increases smoothly in all but the lowest region of symptom severity, but increases more slowly than the item characteristic curve in Item 1 or 3. Expected item scores for this item are generally lower at comparable regions of symptom severity than for Items 1 or 3.

Grandiosity, Item 5, is defined as "Exaggerated self-opinion and unrealistic convictions of superiority, including delusions of extraordinary abilities, wealth, knowledge, fame, power, and moral righteousness" [1]. Figure 4 depicts the option characteristic curves for this item as being generally flatter and less peaked than those of other items. Indeed, the probability of endorsing Options 2, 3, and 5 from Item 5 increase much slower than the corresponding options for Items 1 or 3.

Other important differences between Item 5 (Grandiosity, Figure 4) and Items 1 (Delusions, Figure 2) or 3 (Hallucinatory Behavior, Figure 3) are evident when comparing expected item scores. The expected score is depicted in each of our figures by a broken or dashed line that generally increases smoothly within the severity range from -2 to +2. However, results show that the expected item score for Item 1 (Delusions) and Item 3 (Hallucinatory Behavior) increase much more between -2 and +2 than the expected score for Item 5 (Grandiosity). Indeed, the value of the expected item scores at the midpoint of the sample (0 standard normal quantiles) is 3.5 (right hand abscissa) for Item 1 (Delusions), 3.8 for Item 3 (Hallucinatory Behavior), and only 2.2 for Item 5 (Grandiosity). In this sense, Item 5 assessing Grandiosity is "harder" (much less discriminating) than Items 1 or 3. That is, a score of 3.5 on Item 5 (Grandiosity) is expected only when symptom severity exceeds +1 standard units, which translates to 103 on the Positive Symptom subscale or 36.1 on the entire 30-item PANSS.

#### Mannerisms and posturing

**Figure 5:**
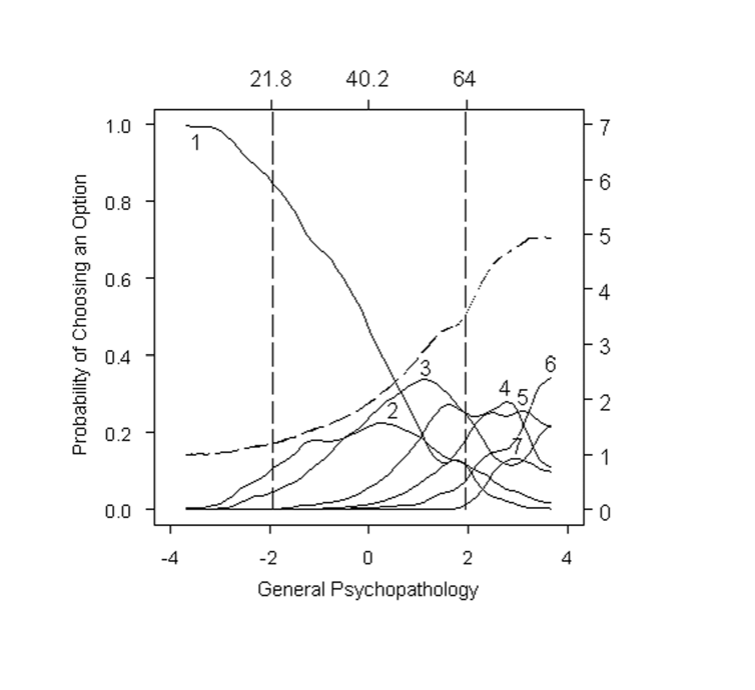
**Option characteristic curves and expected item total score for Item 19: Mannerisms and posturing**. Option characteristic curves (solid lines) and expected item total score (dashed line) are plotted as a function of scores on the General Psychopathology Subscale from the PANNS, expressed as standard normal scores (lower x-axis) and expected total scores (upper x-axis). Option characteristic curves (solid lines) overlap between Options 1 and 2 but generally well differentiated. However, options are generally only endorsed at more severe levels of symptomatology. Option 1 was most likely to be endorsed than any other option, in over half the sample, namely with individuals scoring less than 81 on the 30-item PANSS.

Item 19 from the PANSS assesses Mannerisms and Posturing, which is defined as "Unnatural movements or posture characterized as awkward, stilted, disorganized, or bizarre". Option characteristic curves for this item, Figure 5, tend to increase less quickly than those for Item 1 (Delusions.) As a result, Options 2 through 6 of Item 19 are likely to be endorsed at much higher severity levels than the corresponding options of Item 1. Item 19 (Mannerisms and Posturing) could, therefore, be termed a "hard" item.

### Evaluating total and subscale performance

**Figure 6:**
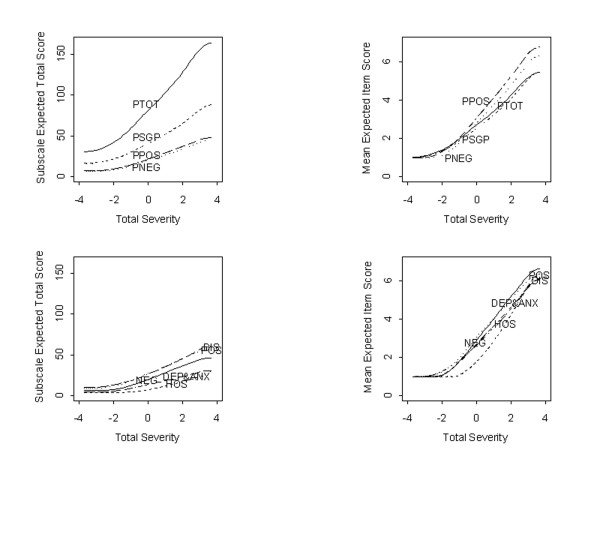
**Expected total scores for subscales from the PANSS**. Results show that the Positive and Negative subscales are more discriminating (i.e., have steeper slopes) than other subscales demonstrating stronger discrimination on a per item basis (top right panel) than the total PANSS Scale.

The ability of various subscales to discriminate among levels of severity was examined by computing and plotting expected subscale totals for the 5 PANSS factor scales of Davis and Chen [9], as well as for the 30-item PANSS scale in total (TOT) and the original General Psychopathology subscale in Figure 6. Results suggest that the Positive and Negative subscales are more discriminating than the General Psychopathology subscale score or the PANSS total score. That is, a 1-unit change in underlying symptom severity corresponds to a greater expected change in per item score (second panel) for the Positive and Negative subscales than for the Full Scale or General Psychopathology subscale.

### Evaluating the appropriateness of symptom remission cutoff scores

**Figure 7:**
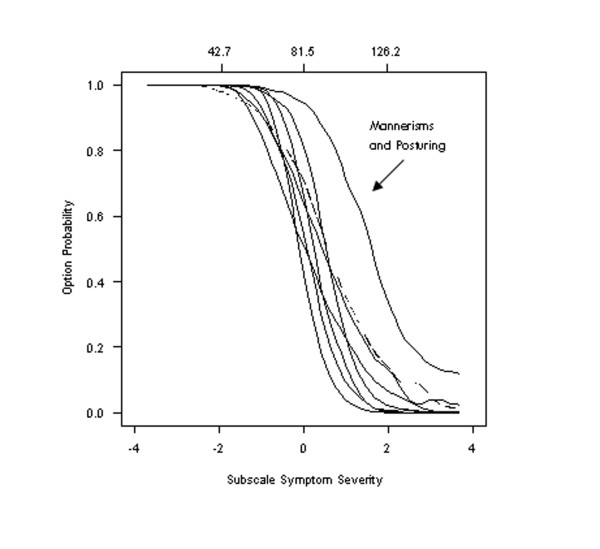
**Option characteristic curves for Item 19: Mannerisms and posturing**. Option characteristic curves describing the probability with ratings of 3 or less are made as a function of symptom severity for items recommended to define symptom remission in patients. Results show that the option characteristic curves for this item will be endorsed at a much more severe level of symptomatology than other items and is therefore redundant or should be revised. The broken line (---) shows the option characteristic curves for a revised Item 19, where remission is defined on the basis of a score less than or equal to 2 (rather than 3).

Andreasen and her colleagues [17] recently recommended that "symptom remission" be defined as achieving scores of *3 or less *on each of 8 core symptoms: Delusions, Unusual Thought Content, Hallucinatory Behavior, Conceptual Disorganization, Mannerisms and Posturing, Blunted Affect, Social Withdrawal, and Lack of Spontaneity. To examine the appropriateness of this common cut-point, we modeled the probability of being rated *3 or less *for each of these 8 symptoms as a function of symptom severity. Specifically, the response characteristic curves for endorsing Option 1, 2, or 3 were computed by summing across individual option characteristic curves. Results show that the probability of obtaining a score of 3 or less does decrease for 7 of the 8 core symptoms. For the "easy" Item 19 (Mannerisms and Posturing), the probability of obtaining a score of 3 or less decreases more slowly over a slightly wider range of symptom severity. This means that a person with a severity score of +2 has a probability of 0.5 of scoring 3 or less on Item 19 (Mannerisms and Posturing). Meanwhile, the probability of scoring 3 or less on any other item drops to less than 0.2 at a severity score of +2 having declined from a probability of 0.5 achieved at a much lower severity score (Figure 7).

As a result, reaching remission criteria for Item 19 will be easier than for the other 7 symptoms and suggests that a threshold of 1 or 2 for Item 19 might be more appropriate.

## Discussion

### Option and item performance

Results of our analyses, summarized in Table 1, confirm that most PANSS items are either *very good *or *good *at assessing the overall severity, particularly items within the Negative Symptom subscale. However, a number of items, largely those from the original General Psychopathology subscale, performed far less well due to difficulties with some options. One of the few, consistent difficulties for a large number of items is the overlap in response characteristic curves for Options 1 and 2. In fact, there may be no region of severity in which Option 2, indicating minimal symptoms, is most likely to be endorsed than Option 1, indicating an absence of symptoms. This result is not unexpected, because the definition of Option 2 in all such items includes "can be at the extreme of normal." This phrasing appears to create greater overlap between Options 1 and 2 than between other adjacent options.

Second, our results also demonstrate overlap between a number of adjacent option characteristic curves. In particular, the Difficulty in Abstract Thinking, Hostility, Uncooperativeness, and Hallucinatory Behavior items display overlap between all items suggesting that they are poorly differentiated. Third, results showed that many options within several "hard" items are endorsed only at much higher levels of severity. For example, Option 1 of Grandiosity was endorsed for more than half of the patients.

### Subscale performance

Our results show that items from the Positive and Negative subscales are generally more discriminating of individual differences in severity than any other of the subscales. In particular, items from the Positive and Negative subscales are more discriminating *on average *than the full 30-item PANSS total and, thus, ***may ***be more sensitive to change than the PANSS total. These 2 subscales might possibly constitute a "mini PANSS" that would be more reliable, require shorter administration and training time, and might even reduce the sample size needed for future schizophrenia research.

### Defining remission criteria

Our detailed analyses of the 8 items currently recommended as markers of remission reveals a problem with using a common threshold of "3 or less." Specifically, Option 3 of Item 19 (Mannerisms and Posturing) may be endorsed at slightly higher levels of severity than any of the other 7 items being considered. As a result, the threshold for remission on Item 19 might be more appropriately set at 1 or 2. Examining the detailed plots of all option characteristic curves for items used to define remission (see Additional file 1) shows that Option 3 on Item 19 is the only option most likely to be endorsed in range of symptom severity >0 (or >81 on the total PANSS). Options 1, 2, and 3 for all other items are most likely to be endorsed in a range of severity less than 81.

## Conclusion

This first item response analysis (IRA) of the PANSS supports the overall sound psychometric properties of the PANSS and demonstrates that most of its items are very good or good at assessing the overall schizophrenia severity. There were a number of items, primarily on the General Psychopathology subscale, that might best be modified and/or scored with fewer levels. The Positive and Negative subscales may also be more sensitive to change than the PANSS total score. On the basis of these results, one might consider rewriting item options so that (a) they are more sensitive to differences at low severity and/or (b) the higher level options are endorsed at lower severity levels. On the other hand, the observed failure to discriminate among individuals in the very lowest regions of severity, such as -4 to -3, might be due to relatively high severity in our sample. Our curves may not be clinically useful with low severity patients. Alternately, one might modify rater training to change option endorsement probabilities. Our results suggest that certain options are not currently being adequately differentiated. Interestingly, one might estimate option characteristic curves at different phases or types of training to evaluate the effectiveness of different types and lengths of training programs.

## Competing interests

The author(s) declare that they have no competing interests.

## Authors' contributions

DS performed the analyses, led the interpretation of the results, and drafted the manuscript. HA-S conceived of the study, participated in its design, the analytical plan, the interpretation of the results, and helped draft the manuscript. JPL and RO participated in the design of the study, the analytical plan, the interpretation of the results, and assisted in drafting the manuscript. All authors have read and approved the final manuscript.

## Pre-publication history

The pre-publication history for this paper can be accessed here:



## Supplementary Material

Additional file 1 Appendix. Option characteristic curves for all PANSS items. Click here for file
