# Supplementary figures and images for: Item response analysis of the Positive and Negative Syndrome Scale
Source: BMC Psychiatry. 2007 Nov 15;7:66. doi: 10.1186/1471-244X-7-66 (PMC2211479; doi:10.1186/1471-244X-7-66)

## Slide 1
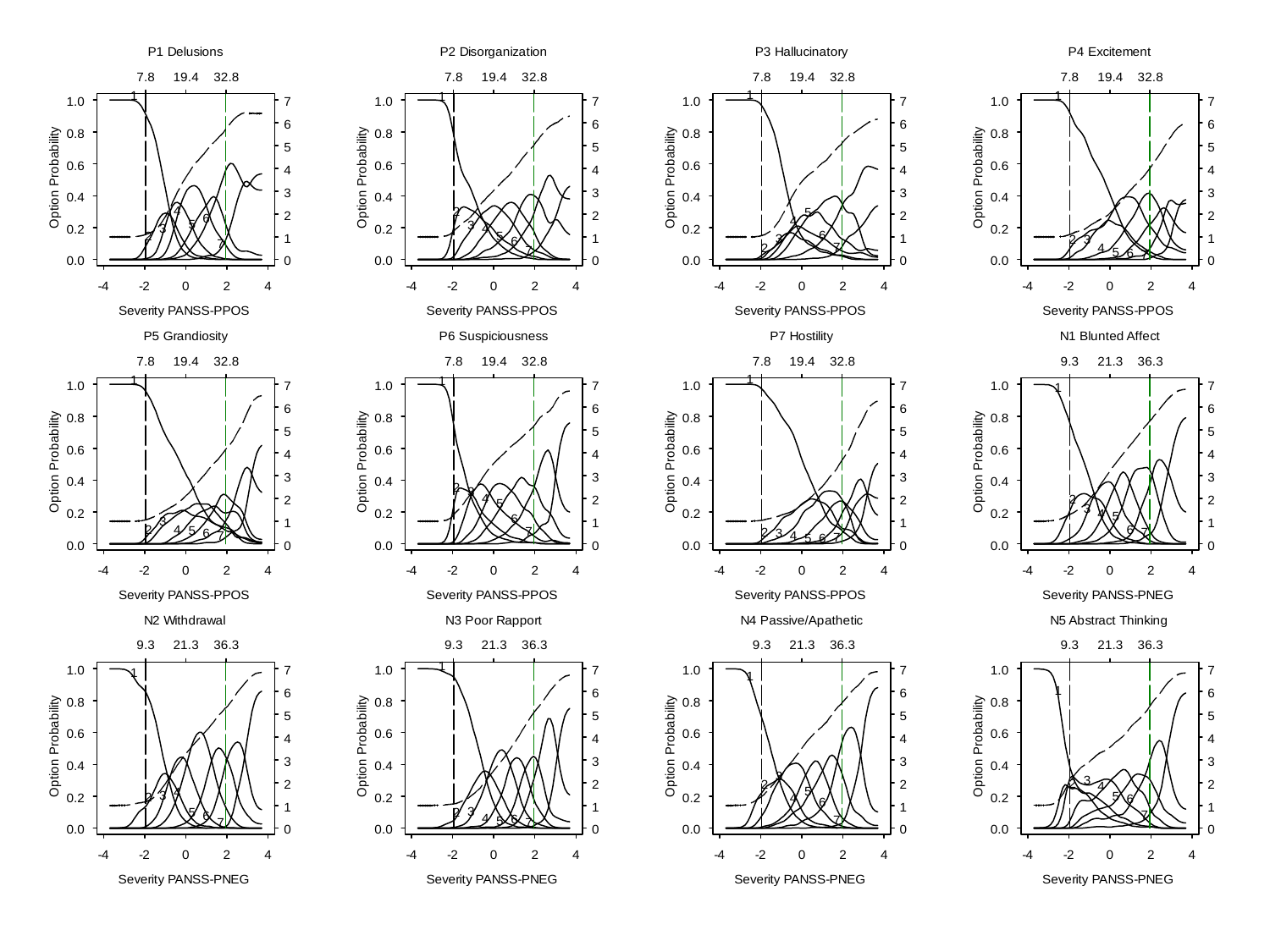

## Slide 2
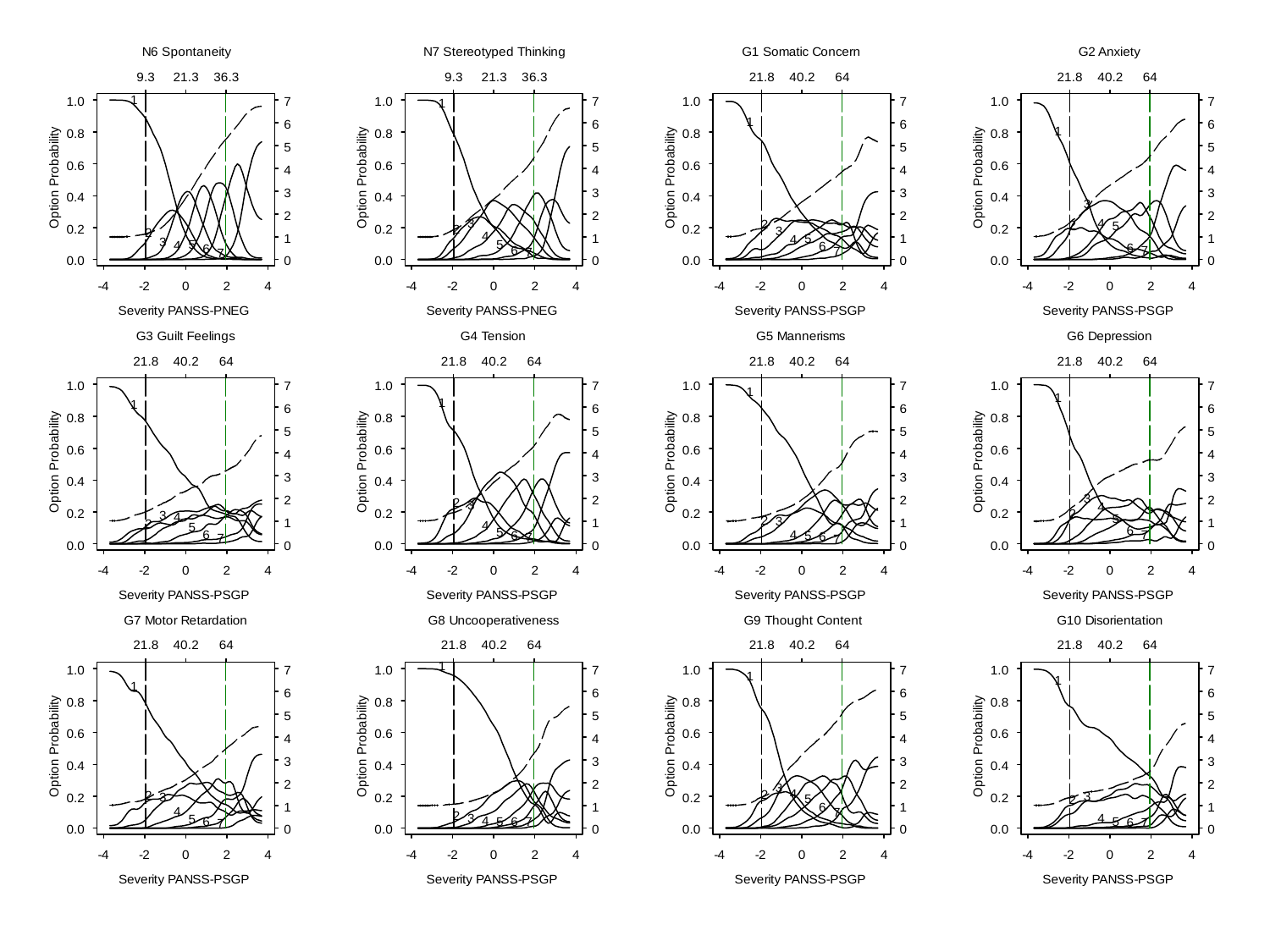

## Slide 3
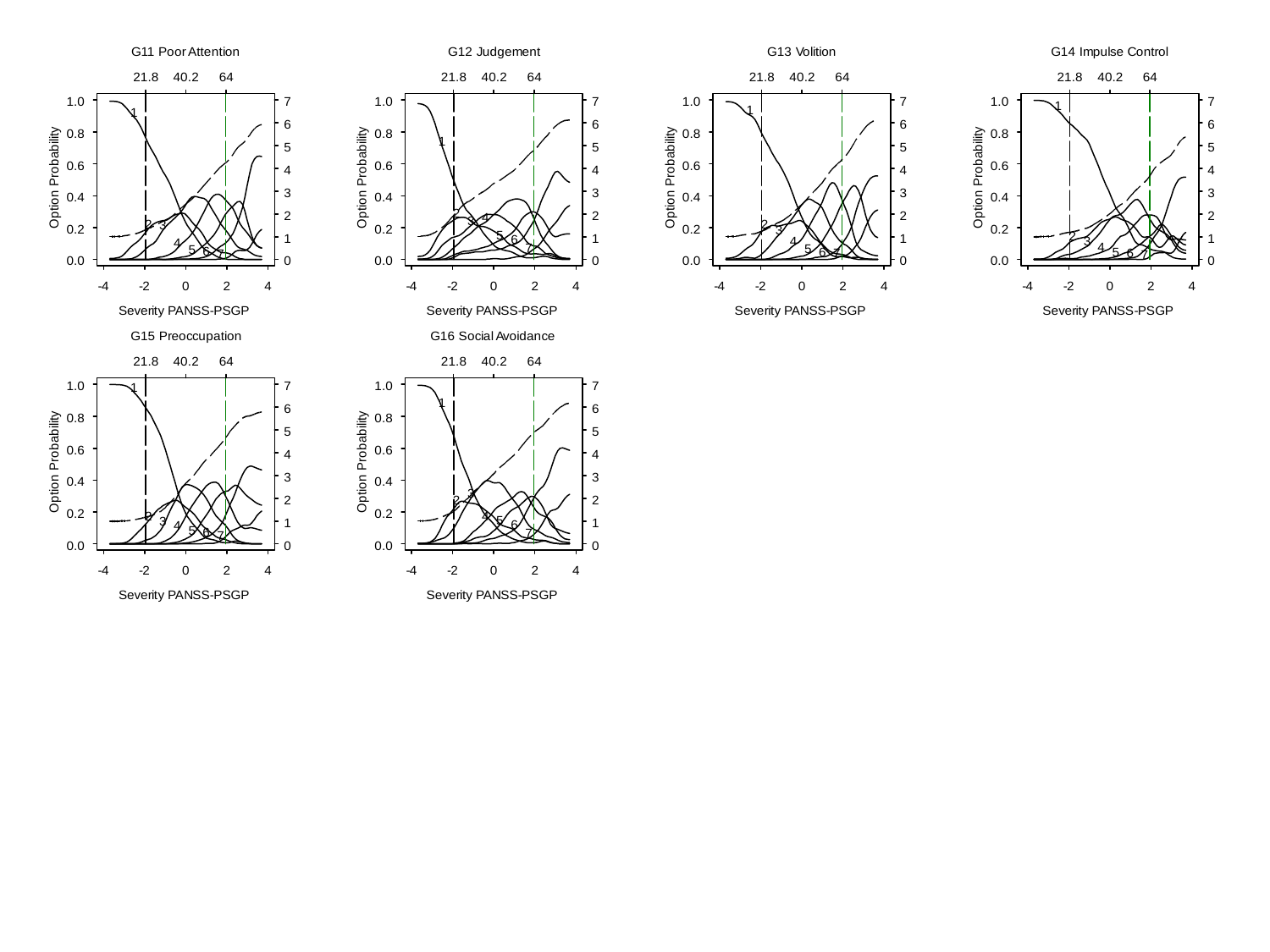

Supplement: Additional file 1 — Appendix. Option characteristic curves for all PANSS items. [file 1471-244X-7-66-S1.ppt]
